# Resting-state brain activity dysfunctions in schizophrenia and their associations with negative symptom domains

**DOI:** 10.1192/j.eurpsy.2023.1161

**Published:** 2023-07-19

**Authors:** P. Pezzella, G. M. Giordano, L. Fazio, L. Giuliani, A. Mucci, P. Bucci, M. Amore, P. Rocca, A. Rossi, A. Perrottelli, E. Caporusso, A. Bertolino, S. Galderisi, M. Maj

**Affiliations:** 1 Department of Psychiatry, University of Campania “Luigi Vanvitelli”, Naples; 2 LUM - Libera Università Mediterranea “Giuseppe Degennaro”, Bari; 3 Department of Neurosciences, Rehabilitation, Ophthalmology, Genetics and Maternal and Child Health, Section of Psychiatry, University of Genoa, Genoa; 4 Department of Neuroscience, Section of Psychiatry, University of Turin, Turin; 5 Department of Biotechnological and Applied Clinical Sciences, Section of Psychiatry, University of L’Aquila, L’Aquila; 6 Department of Basic Medical Science, Neuroscience and Sense Organs, University of Bari ‘Aldo Moro’, Bari, Italy

## Abstract

**Introduction:**

Negative symptoms represent a fundamental aspect of schizophrenia: they have a substantial impact on patients’ real-life functioning and do not respond satisfactorily to currently available treatments. Therefore, a better understanding of the pathophysiological mechanisms underlying these symptoms could favor the development of new treatments.

To date, the most validated pathophysiological hypothesis indicates an association between the Motivational domain (consisting of avolition, anhedonia and asociality) and alterations in the neuronal circuits involved in motivation. The Expressive Deficit domain (consisting of blunted affect and alogia) would be subtended by widespread alterations of cortical connectivity and associated with impaired neurocognition, social cognition, and the presence of neurological soft signs.

**Objectives:**

The aim of the present study is to examine the neurobiological correlates of the two domains of negative symptoms, starting from the brain areas that have been most commonly found in the literature to be associated with negative symptoms.

**Methods:**

Resting-state (rs) fMRI data were acquired in 62 subjects with schizophrenia (SZ) and 46 healthy controls (HC). The two negative symptom domains were assessed using the Brief Negative Symptom Scale. In addition, the following assessment tools were used: the Positive and Negative Syndrome Scale for the assessment of positive symptoms and disorganization, the Calgary Depression Scale for Schizophrenia for depression and the St. Hans Rating Scale for extrapyramidal symptoms. The study of the possible relationships between rs-brain activity and the negative symptoms domains was conducted through partial correlations, checking for possible confounding factors (positive, depressive, extrapyramidal symptoms and disorganization).

**Results:**

The SZ, compared to the HC, showed higher rs-brain activity of the right inferior parietal lobule and of the right temporoparietal junction and lower rs-brain activity of the right dorsolateral prefrontal cortex, bilateral anterior dorsal cingulate cortex, bilateral ventral caudate and bilateral dorsal caudate. Furthermore, in the group of patients, the rs-brain activity of the left ventral caudate showed a moderate negative correlation with the Expressive deficit domain (r = -0.401; p = 0.003), but not with the Motivational domain.

**Conclusions:**

The results of the present study, in line with the literature, demonstrated how the two domains of negative symptomatology are subtended by different pathophysiological mechanisms. Given the role played by the ventral caudate in neurocognitive processes, these results are in line with the hypothesis that Expressive deficit may have a common etiopathogenesis with cognitive deficits. A better understanding of the neurobiology of negative symptoms could foster the development of innovative treatment strategies targeting the two negative symptom domains.

**Disclosure of Interest:**

None Declared

